# Boron neutron capture therapy for melanoma: recent advances and future prospects

**DOI:** 10.3389/fonc.2025.1485207

**Published:** 2025-09-01

**Authors:** Yuanyang Sun, Yun Wang, Shukun Mu, Xiaofeng Wu, Suchun Yu, Zhongming Wang

**Affiliations:** ^1^ School of Health Science and Engineering, University of Shanghai for Science and Technology, Shanghai, China; ^2^ Department of Radiation Oncology, Shidong Hospital, Shidong Hospital Affiliated to University of Shanghai for Science and Technology, Shanghai, China; ^3^ Department of Pharmacy, Shidong Hospital, Shidong Hospital Affiliated to University of Shanghai for Science and Technology, Shanghai, China

**Keywords:** boron neutron capture therapy, melanoma, clinical outcome, boron delivery agent, accelerator-based neutron source

## Abstract

Boron neutron capture therapy (BNCT) is a unique radiotherapy modality that targets and destroys selectively tumor cells that have absorbed boron, while leaving surrounding healthy cells unharmed. Over the course of the last nearly 40 years, several clinical studies of BNCT for melanoma have been conducted in many countries. The results of clinical studies are encouraging, suggesting that BNCT may be a potentially effective method for treating melanoma. In this work, we review the outcomes of clinical study of BNCT for melanoma. Moreover, we provide a concise overview of advancements in accelerator-based neutron source and boron delivery agent (BDA) applied to BNCT for melanoma. Finally, we discuss the areas for further research focus regarding BNCT for melanoma.

## 1 Introduction

Melanoma, a malignant tumor that arises from the malignant transformation of melanocytes, can occur in various tissues, including the skin, mucous membranes, and soft meninges (1). The incidence of melanoma is on the rise worldwide in recent years, particularly prevalent in the white population. The cutaneous melanoma accounts for most of all melanoma diagnoses in whites (more than 90%). The incidence of melanoma in other races has also increased, with a higher prevalence of acral melanoma noted (2). Currently, the standard treatment for nonmetastatic melanoma is surgical resection with stage-appropriate postoperative adjuvant therapy (3). However, surgical resection is highly invasive, especially for senile patients, and may result in various functional and psychological issues. When extensive surgical resection is not feasible, other treatments including chemotherapy, immunotherapy, targeted therapy and particle therapy can be utilized for local control (4–6). BNCT can destroy selectively tumor cells while minimizing damage to nearby healthy cells. In comparison with conventional photon radiotherapy, BNCT induces tolerable toxicity in dose-limiting tissues and improves the quality of life for patients with only one or two applications (7). While BNCT has been investigated for a range of malignancies, melanoma presents a distinct opportunity owing to its unique melanin biosynthesis pathway and amino acid transport properties (6). The initial human clinical trial of BNCT for melanoma took place in Japan in 1987 (8). The tumor lesion showed effective control and did not develop any metastasis following treatment. Subsequently, clinical studies of BNCT for melanoma have been conducted in many countries, and the outcomes have shown encouraging tumor response rates. Recent years, researchers have focused significantly on advancing accelerator-based neutron source and novel BDAs for BNCT for melanoma. Clinical studies involving accelerator-based BNCT (AB-BNCT) for various malignant tumors, including melanoma, are currently being actively conducted. Moreover, several novel BDAs applied to BNCT for melanoma have been created in recent years. These BDAs exhibit better tumor targeting capacity and boron loading capacity than conventional boron delivery agents, showing great therapeutic potential in BNCT for melanoma. In this article, we introduce the principle of BNCT and review the clinical outcomes of BNCT for melanoma. We also introduce the recent advances in BNCT for melanoma, with an emphasis on the AB-BNCT and novel BDAs. Finally, we discuss the further research directions of BNCT for melanoma.

## 2 The principle of BNCT

The concept of BNCT was initially proposed by American astrophysicist Gordon Locher in 1936. BNCT employs nuclear reactions that take place between boron and neutron to exert antitumor effects. Successful BNCT relies on two main factors (1): sufficient boron must be selectively delivered to tumor cells with much less to normal cells by specific BDA, and (2) the appropriate number of neutrons must be provided to trigger nuclear reactions. When tumor cells containing boron-10 (^10^B) are exposed to neutron beam, ^10^B undergoes the capture reaction, transforming into unstable boron-11 (^11^B). Subsequently, ^11^B undergoes the fission reaction, radiating alpha particles (^4^He) and recoil particles (^7^Li) (9). ^4^He and ^7^Li are both charged particles that exhibit high linear energy transfer (LET) and relative biological effectiveness (RBE). These particles can cause damage that is difficult to repair on cells by inducing DNA double-strand breaks, ultimately resulting in apoptosis or necrosis (10). Interestingly, the damage caused by these particles is restricted to cells that contain boron, as their path lengths in tissue are short (5-9 µm). Therefore, the lethal damage caused by BNCT primarily targets tumor cells containing high concentrations of boron. Fernanda et al. (11) evaluated the impact of BNCT on melanoma cells and normal melanocytes. Following BNCT, normal melanocytes showed minimal cell death and free radical production without any morphological change. On the contrary, melanoma cells showed notable alterations in the extracellular matrix and a decrease in cyclin D1 levels. Moreover, BNCT resulted in a decrease in the mitochondrial membrane potential and a significant increase in cleaved caspase-3 levels in melanoma cells, suggesting that BNCT induced both necrosis and apoptosis in melanoma cells. These findings indicated that BNCT had a lower toxicity to normal melanocytes while effectively eliminating melanoma cells via multiple signaling pathways, such as arresting the cell cycle, altering the extracellular matrix, and triggering the apoptosis.

## 3 Clinical outcomes

### 3.1 Japan groups

Mishima et al. (8) reported the first application of BNCT for cutaneous melanoma in 1987. The tumor lesion involved the entire right first toenail and periungual skin. Neutron activation analysis was utilized to assess the boron accumulation in tissues. All 10 perilesional points, situated 4 cm away from the edge of the tumor, received an injection of 640 mg of BPA. The metastasis faded clearly, and the clinical symptoms showed considerable remission around 8 months after BNCT. The metastasis almost disappeared entirely with no signs of recurrence 9 months after BNCT. Only slight redness and dry scaling of the skin were observed after BNCT. Subsequently, Fukuda et al. (12) administrated BNCT with BPA (170 - 200mg/kg) to 22 melanoma patients from 1987 to 2001. The rates for complete response (CR) and partial response (PR) were 73% and 23%, respectively. The response rate for patients with non-nodular melanoma was 94%. However, the response rate for nodular melanoma was significantly lower, at 17%, in contrast to non-nodular melanoma. The skin injuries of 16 patients were within acceptable limits and did not necessitate any medication. The skin injuries of 3 patients required minimal medication and healed in a matter of months. The skin injuries of the remaining 3 patients were relatively severe, with skin ulcers and necrosis, requiring treatment with skin graft. Moreover, Hiratsuka et al. (13) administrated BNCT with BPA to 8 patients with cutaneous melanoma from 2003 to 2014. All tumor lesions were primary and located on the soles of the feet or face. All patients had no local lymph node metastasis or distant metastasis. Out of the patients treated, 6 achieved CR accounting for 75%, while 2 had PR accounting for 25%. There were no reports of serious adverse events such as skin ulcers or necrosis. During the long-term follow-up, 3 patients died of pneumonia or natural causes, while the remaining 5 patients showed no signs of disease. Morita et al. (14) reported the outcomes of 4 patients with melanoma undergone BNCT. The lesions of 2 patients with cutaneous melanoma were located on the heel, 1 patient with mucosal melanoma was located on the nasal cavity, and another patient was located on the vulva and vagina. After BNCT, 2 patients with cutaneous melanoma and 1 patient with nasal mucosal melanoma showed CR, while another patient showed PR without any signs of recurrence. Normal tissue damage in all patients was acceptable. Hiratsuka et al. (15) administered BNCT with BPA-fructose complex (BPA-F) to 1 patient with vulvar melanoma and 3 patients with EMPD. All patients showed CR 6 months following BNCT, and none showed signs of recurrence during long-term follow-up (up to 6.9 years). The most serious normal tissue injuries were moderate skin erosion, which fully healed after 4 months. Hiroshi et al. (16) reported the outcomes of the first case of acral melanoma received AB-BNCT. The patient showed PR 6 months after BNCT and CR after 12 months. And the patient only experienced minor skin injuries such as radiation dermatitis, skin hyperpigmentation, dry skin, and edema, which relieved without any treatment. During the 12-month follow-up period, the patient did not show any signs of recurrence and did not experience any high-grade adverse events.

### 3.2 Other groups

In 2004, Gonzalez et al. (17) reported the first application of BNCT for cutaneous melanoma in Argentina. Following treatment, 21 of 25 nodules showed CR after 8 weeks. The grade 1 acute skin reaction was observed 1 day after BNCT and relieved after 8 weeks. Menendez et al. (18) administrated BNCT to 7 patients with melanoma with multiple subcutaneous skin metastases from 2003 to 2007. All patients received intravenous administration of 14 g/m^2^ of BPA-F. The overall response rate (CR+PR) based on lesions was 69.3%. The toxicity level was acceptable, with 30% of evaluable areas exhibited ulceration. No signs of disease progression were observed during the follow-up period. From 1994 to 1996, the MIT group (19) conducted a study of BNCT for 4 patients with acral melanoma. Following BNCT, 1 patient showed CR, and 2 patients showed PR. The tumor response of another patient could not be evaluated because the residual lesion was resected for biopsy following treatment. In 2016, Yong et al. (20) reported the outcomes of first case of melanoma received BNCT with BPA-F (350 mg/kg) in China. The lesions were located on the bottom and heel of the left foot. The fluctuations in boron levels in the bloodstream were assessed by conducting several blood draws from patients over a period of time. The IHNI - 1, which is based on a miniature neutron source reactor developed and built by the China Institute of Atomic Energy, served as the neutron source. The patient showed CR after BNCT, and no severe treatment-related adverse events were observed during the two years of follow-up.

A summary of clinical outcomes of BNCT for melanoma is provided in **Table 1**.

**Table 1:** Clinical outcomes of BNCT for melanoma.

Time	Phase	Drug	Case	ORR	High-grade toxicity	Citation
1987-2001	I/II	170–210 mg BPA/kg	22	CR:73%, PR:23%	6/22 (27%)	(12)
1994-1996	I/II	BPA^*^	4	CR:25%, PR:75%	Not available	(19)
2003-	II	160 mg BPA/kg	4	CR:75%, PR:25%	0/4	(14)
2003-2007	I/II	14 g BPA/m^2^	7	CR+PR: 69.3%	3/7 (43%)	(18)
2003-2014	II	200 mg BPA-F/kg	8	CR:75%, PR:25%	0/8	(13)

ORR` objective response rate; CR, complete response; PR, partial response; BPA, boronophenylalanine; BPA-F, BPA-fructose complex; BPA^*^, The dosage of BPA is not described in literature.

## 4 Recent advances in BNCT for melanoma

### 4.1 From reactor to accelerator

Neutron can be categorized as the thermal neutron, epithermal neutron, and fast neutron based on their energy levels. The neutron commonly used in BNCT practice is thermal neutron or epithermal neutron. The ideal neutron source in BNCT should possess the following characteristics (1): the neutron produced should be predominantly thermal neutron or epithermal neutron (2); the gamma ray produced should be as less as possible; (3) the neutron flux density should be greater than 10^9^ n/cm^2^/s; (4) the maximum neutron flux produced should be within tumor region enriched boron. The currently available neutron sources primarily include reactor and accelerator. Neutrons produced by reactor are processed to obtain high stability, high intensity and high purity thermal neutron beam or epithermal neutron beam. However, reactors are commonly constructed in non-medical settings that are far distant from urban areas, and their maintenance costs are high, significantly limiting the clinical application of BNCT. Accelerator, as another available neutron source, can produce neutron by accelerating proton or deuterium hitting the lithium or beryllium metal target (21). Herrera et al. (22) performed a dosimetry analysis comparing reactor-based BNCT (RB-BNCT) with AB-BNCT in the treatment of different malignant tumors. The results revealed that the lowest weighted doses for patients with brain tumor and head and neck cancer receiving AB-BNCT were greater than 30 Gy and 14 Gy, respectively, which is consistent with the results from RB-BNCT. The minimum tumor doses for patients with melanoma receiving AB-BNCT were equal to or greater than those obtained with RB-BNCT. These findings demonstrated the potential applicability of AB-BNCT for superficial and deep tumors. In 2009, Sumitomo Heavy Industries collaborated with Kyoto University to successfully develop a neutron source based on a cyclotron (23). In 2012, Kyoto University conducted the initial clinical trial of AB-BNCT. Additionally, Cancer Intelligence Care Systems, Inc. based in Tokyo, Japan, has developed an AB-BNCT system known as CICS - 1. They carried out a clinical trial to assess the safety and effectiveness of AB-BNCT utilizing CICS - 1 for treating cutaneous angiosarcoma and melanoma (24). In this trial, 10 patients (9 scalp angiosarcoma, 1 forefinger malignant melanoma) received AB-BNCT between November 2019 and April 2022. AB-BNCT exhibited a good safety profile and a high rate of response in these patients. Only one patient experienced a grade 3 asymptomatic increase in serum amylase levels. The best overall response rate was 70%, with a median tumor reduction rate of 77.5%. The tumor lesion of melanoma patient kept shrinking after 180 days, ultimately achieving a CR at 360 days. The 2-year PFS, LPFS, and OS rates for all patients were 15.0%, 40.0%, and 90.0%.

In contrast to reactor, accelerator provides many benefits, including that it avoids the production of large amounts of radioactive waste, it costs less, and it can be installed in hospitals due to its small footprint (25). However, increasing the neutron flux remains the problem that AB-BNCT needs to overcome. Currently, multiple AB-BNCT projects and related clinical studies have been initiated around the world (26, 27). Many countries such as Japan, Argentina, China, Finland, South Korea, Great Britain, Italy and Russia are building or planning the BNCT centers, which will increase significantly the number of tumor patients who can undergo BNCT (28). Based on these BNCT centers, further research can be conducted to facilitate the clinical application of BNCT.

### 4.2 Novel BDAs

Currently, BPA is the most common BDA in BNCT for melanoma. Although BPA shows encouraging results in terms of clinical effectiveness, it still has many drawbacks, including required high dosage, poor water solubility and limited tumor targeting ability, which limits the therapeutic effect of BNCT for melanoma to a certain extent (29). With the continuous development of synthetic technology and oncologic pathology, various novel BDAs applied to BNCT for melanoma have emerged.

#### 4.2.1 BDAs made of nanoparticles

Nanoparticles exhibit prolonged circulation and passive accumulation at pathological sites such as tumors, metastases and inflammation sites (30). This phenomenon can be explained by the existence of leaky vasculature and a large number of phagocytes (31). Additionally, nanoparticles can be modified with targeting ligands to enhance their interaction with and absorption by specific cells or tissues (32). These characteristics make nanoparticles ideal for use in drug delivery (33). At present, boron-containing nanoparticles have attracted significant interest as the promising drug delivery systems for BNCT.

It has been demonstrated that the overexpression of sialic acids is strongly associated with the aggressiveness and metastatic capabilities of various tumors (34). Phenylboronic acid (PBA), which has excellent water solubility and low systemic toxicity, can enhance endocytosis by binding with sialic acid receptors (35). PBA-sialic acid has been identified as a ligand-receptor combination for targeting tumor cells. In addition, PBA has the potential to serve as a BDA that targets the nucleus, as it can assist in transporting boron to the nucleus by binding with importin (36). A recent research evaluated the potential of PBA as a nuclear-targeting BDA (37). The boron concentration of PBA in melanoma cells reached 74.47 ± 5.64 ng/10^6^ cells, which was superior to that of sodium borocaptate (BSH). Moreover, the boron concentration of PBA in the nucleus reached 45.77 ± 5.64 ng/10^6^ cells, exceeding than that in the cytoplasm. Kim et al. (38) synthesized a polymeric nanoparticle decorated with PBA (Nano^PBA^) to serve as a BDA that targets sialic acid. Nano^PBA^ exhibited dual functions of neutron capture and tumor cell targeting. It can quickly attach to the tumor cell membrane, showing superior effectiveness *in vitro* cellular uptake into cancer cells when compared to BPA-F. *In vivo* experiments demonstrated that Nano^PBA^ achieved excellent antitumor effects in melanoma-bearing mice, even at a dose 100 times lower than BPA-F, thanks to its effective targeting of tumors and preferential delivery to the intracellular region near the nucleus.

Boronated porphyrins offer many benefits, including (1): significant accumulation and prolonged retention in tumor cells (2); great water solubility and minimal systemic toxicity (3); high percentage of boron content; and (4) potential imaging properties (39). Shi et al. (40) synthesized a boronated porphyrin nanocomplex (BPN) by coating boronated porphyrins with biocompatible poly (lactide-co-glycolide)-monomethoxy-poly (ethylene-glycol) (PLGA−mPEG) micelles. BPN can facilitate the delivery of boron by incorporating tetraboronated porphyrin (TBPP) with PLGA−mPEG micelles. And the micelles prevent the direct interaction between BPN and blood cells, which reduces toxicity and enhanced tumor targeting. Moreover, BPN achieves the tumor location and visualization of boron concentration in mice via fluorescence imaging and PET imaging. The experiments also demonstrated that BPN showed remarkable anti-tumor effects in melanoma-bearing mice, indicating that BPN has great potential in imaging-guided BNCT for melanoma.

Detonation nanodiamonds (DNDs) are biologically inert nanoparticles that are covalently modified by oxygen-containing functional groups on the surface through robust processes (41). DNDs containing fluorescent color centers like nitrogen vacancy and silicon vacancy centers have demonstrated significant promise in biosensing, imaging, and theragnostic (42, 43). In addition, poly(glycerol) (PG) functionalization is employed in nanomaterials in the biomedical field because of its hydrophilicity (44). Nishikawa et al. (45) designed nanodrugs for BNCT and evaluated their cellular uptake and anti-tumor efficacy in murine melanoma cells. These nanodrugs are made up of poly(glycerol) functionalized detonation nanodiamonds (DND-PG) serving as a hydrophilic nanocarrier, a boron cluster moiety (^10^B_12_H_11_^2-^) providing the boron source, and either PBA or RGD peptide acting as the active targeting component. Transmission electron microscopy observations of melanoma cells treated with nanodrugs showed that ^10^B_12_H_11_^2-^ moiety facilitated the cellular uptake of melanoma cells significantly more than the active targeting moieties PBA and RGD. Moreover, the neutron irradiation experiments showed that nanodrugs exhibited good efficacy, with minor differences between those that included an active targeting component and those that did not.

Polymer micelles with obvious core-shell structure exhibit great potential as the BDA because of their outstanding biocompatibility and biodegradability (46). The hydrophilic outer layer of polymer micelles can generate steric repulsion, which conceals the encapsulated drug and helps it avoid detection by the mononuclear phagocytic system, ultimately avoiding the rapid elimination of the drug (47). In addition, the dimensions of polymer micelles usually surpass the renal filtration limit, allowing them to remain in the bloodstream for a longer period (48). Fu et al. (49) synthesized boron-rich polymer micelles via atom transfer radical polymerization (ATRP) and investigated their potential in BNCT. *In vitro* experiments using melanoma cells revealed that polymer micelles demonstrated outstanding biocompatibility, maintaining cell viability above 92% at all tested concentrations. And these micelles showed a 38 times greater cellular boron accumulation in comparison to BPA. The mice that received BNCT utilizing these polymer micelles exhibited a significant postponement in tumor development. Another study also developed the boron-rich block copolymer micelles and evaluated their boron uptake and anti-tumor efficacy (50). *In vivo* studies conducted on melanoma-bearing mice showed that these micelles resulted in an 8-fold increase in boron absorption compared to BPA, which in turn delayed tumor growth. Moreover, they found that BNCT combined with anti-PD-L1 further delayed tumor growth and enhanced immune response in melanoma-bearing mice.

#### 4.2.2 BDAs made of amino acids

The boron-containing amino acid derivatives can effectively target tumor through the amino acid metabolism pathway and show significant promise as BDAs in BNCT for melanoma. Li et al. (51) developed a boron-derived tyrosine (fluoroboronotyrosine, FBY) as a BDA and PET tracer. FBY has a chemical structure that closely resembles that of natural tyrosine and exhibits greater stability compared to BPA. The PET scans revealed that ^18^F-labeled FBY accumulated abundantly in melanoma cells and accumulated obviously at low levels in normal tissues. Moreover, they assessed the relationship between the PET images and the distribution of boron concentration. The detected linear relationship suggested that boron concentration could potentially be estimated using PET imaging for FBY-BNCT. The neutron irradiation experiments showed that FBY-BNCT prolonged significantly the survival of melanoma-bearing mice without causing obvious systemic toxicity.

Several novel BDAs are derived from BPA. Kondo et al. (52) synthesized a positional isomer of BPA, 3-borono-L-phenylalanine (3-BPA). The water solubility of 3-BPA was significantly better than that of BPA. It is precisely because of its excellent water solubility that it allowed for the removal of solubilizer sugars, unlike BPA, while also possessed tumor targeting ability equivalent to that of BPA. The fluorocarbon bonds can enhance drug uptake in tumor cells by improving the permeability of cell membrane and the pharmacokinetics of drug (53). Ding et al. (54) synthesized fluorinated BPA derivatives featuring various fluorine groups and assessed their effectiveness in delivering boron in models of melanoma and glioblastoma. The results showed that fluorinated BPA derivatives exhibited excellent biocompatibility and low systemic toxicity. And the boron accumulation in tumor cells of these fluorinated BPA derivatives was superior to those of BPA-F in melanoma and glioblastoma models.

## 5 Discussion

BNCT is a promising radiotherapy modality and has been investigated extensively in many malignant tumors (55). Advancements in accelerator-based neutron source technology have expanded significantly the scope of services of BNCT. The clinical outcomes of cutaneous melanoma treated with AB-BNCT also have indicated that AB-BNCT had favorable effectiveness and safety in the treatment of melanoma. Moreover, although recently reported novel BDAs improved significantly the boron accumulation and T/N ratio in melanoma in preclinical research, their full potential can only be determined through assessment in clinical trials for BNCT. In addition to the development of novel BDAs, researchers also focus on exploring new administration strategy to improve the T/N ratio. Microneedles (MNs) are patches made up of arrays of tiny needles that can deliver drugs (56), vaccines (57), nanoparticles (58), proteins (59) and antibodies (60) to targeted sites via transdermal route of administration. The administration mode based on MNs has advantages of painless and non-invasive, and is expected to enhance local accumulation of drugs (61). Li et al. (62) developed a new transdermal administration mode based on dissolving microneedles, PVA/BPA-F MNs, and evaluated its effect in a melanoma-bearing mouse model. Owing to the elevated BPA-F concentration encapsulated within MNs, PVA/BPA-F MNs enhanced significantly the delivery of BPA-F to melanoma cells. The T/N ratio was also improved, surpassing greatly that of conventional intravenous injection. The animal experiments showed that BNCT utilizing PVA/BPA-F MNs suppressed significantly tumor growth. However, the transition of MNs technology from research labs to clinical settings still faces many challenges at present, including several parameters affecting MNs insertion, biocompatibility, loading capacity, safety and cost (63). While numerous preclinical studies have assessed the effects of MNs, there is still a lack of corresponding clinical research. Further clinical studies are required to confirm the effectiveness and safety of MNs in clinical application.

Moreover, researchers have explored the combination of BNCT and other clinical approaches to improve therapeutic effects. For example, combining BNCT with immunotherapy (64), chemotherapy (65), photothermal therapy (66) and photodynamic therapy (67) have been investigated actively for the treatment of various tumors. Especially immunotherapy, it is one of the most effective treatment strategies for melanoma. The current discussion on BNCT combined with ICIs is primarily based on preclinical studies in localized tumor models. However, recent preclinical evidence indicates that BNCT can induce abscopal effects and enhance systemic antitumor immunity when combined with ICIs (68). This suggested that BNCT may hold promise not only for local control but also for systemic disease in advanced melanoma with metastases, where immune evasion and radioresistance are major challenges. Combination treatment can integrate advantages of different treatments and overcome limitations of BNCT and other treatments to achieve ideal therapeutic effect. It is necessary to further explore the potential benefit of combined treatment of BNCT with other treatments in treating melanoma.

Moreover, several researchers also utilize radiosensitizer to improve the effectiveness of BNCT in destroying melanoma cells. Radiosensitizers are normally chemicals or drugs that increase the effectiveness of radiotherapy on tumor cells by facilitating DNA damage and generating free radicals. Ideal radiosensitizers usually possess the following characteristics (69) (1): the ability to enhance the effect of radiation (2); excellent tumor targeting ability and biocompatibility (3); low toxicity and rapid excrete from the body. Valproic acid (VPA), a histone deacetylase inhibitor, has been shown to improve the effectiveness of radiotherapy in treating tumors (70). Lai et al. (71) evaluated the enhanced effect of destroying melanoma cells induced by combination treatment of VPA and BNCT. They found that combined treatment of BNCT and VPA exacerbated and perturbed DNA DSBs in melanoma cells and suppressed significantly the growth of melanoma cells. It is necessary to conduct further clinical study to verify the potential of radiosensitizer in BNCT.

Finally, although the outcomes of completed clinical studies of BNCT for melanoma show that BNCT can achieve excellent tumor control with low systemic toxicity, related clinical studies conducted are still scarce so far. Only by obtaining more high-quality clinical study evidence can we facilitate effectively the application of BNCT for melanoma. Moreover, making a model for each patient to help with personalized BNCT also is an area of focus. With the further perfection of the translating regulation and clinical practice, BNCT will have broad application prospect for treating melanoma based on its unique mechanism.

## 6 Conclusion

BNCT is an emerging and promising tumor treatment that can specifically target and eliminate tumor cells while preserving the surrounding healthy cells. The outcomes of clinical studies of BNCT for melanoma have demonstrated that BNCT is a promising approach for treating melanoma. AB-BNCT provides many benefits compared with conventional RB-BNCT, expanding the scope of BNCT services and ultimately establishing a sound foundation for the clinical application of BNCT to treat melanoma. Recently reported novel boron delivery agents and administration mode exhibit great potential in improving the boron accumulation and T/N ratio. However, further clinical studies are needed to fully evaluate their effectiveness and safety. Moreover, more large sample clinical trials are required to validate comprehensively the effectiveness and safety of BNCT for melanoma. With the continuous advancement of pharmaceutical research, radiation device and clinical study, BNCT will contribute more to the treatment of melanoma.
